# A meta-analysis of leaf gas exchange and water status responses to drought

**DOI:** 10.1038/srep20917

**Published:** 2016-02-12

**Authors:** Weiming Yan, Yangquanwei Zhong, Zhouping Shangguan

**Affiliations:** 1 State Key Laboratory of Soil Erosion and Dryland Farming on the Loess Plateau, Northwest A&F University, Yangling, Shaanxi 712100, P.R. China

## Abstract

Drought is considered to be one of the most devastating natural hazards, and it is predicted to become increasingly frequent and severe in the future. Understanding the plant gas exchange and water status response to drought is very important with regard to future climate change. We conducted a meta-analysis based on studies of plants worldwide and aimed to determine the changes in gas exchange and water status under different drought intensities (mild, moderate and severe), different photosynthetic pathways (C_3_ and C_4_) and growth forms (herbs, shrubs, trees and lianas). Our results were as follows: 1) drought negatively impacted gas exchange and water status, and stomatal conductance (g_s_) decreased more than other physiological traits and declined to the greatest extent in shrubs and C_3_ plants. Furthermore, C_4_ plants had an advantage compared to C_3_ plants under the same drought conditions. 2) The decrease in g_s_ mainly reduced the transpiration rate (T_r_), and g_s_ could explain 55% of the decrease in the photosynthesis (*A*) and 74% of the decline in T_r_. 3). Finally, gas exchange showed a close relationship with the leaf water status. Our study provides comprehensive information about the changes in plant gas exchange and water status under drought.

Drought is considered to be one of the most devastating natural hazards and is a pervasive climate phenomenon across the world. It has been predicted that drought will become increasingly frequent and severe due to climate change in the interior of numerous continents^1^,^2^. Additionally, global warming may offset any modest increases in precipitation by increasing evapotranspiration, which will eventually result in further decreases in soil moisture^3^. Decreased soil moisture will inhibit plant growth (even causing mortality) and negatively affect ecosystems^4^,^5^.

Water is a key resource for plant growth and survival and can shape the nature of plant–plant interactions in a wide range of terrestrial ecosystems. Plants perceive and rapidly respond to alterations (even small ones) in water status via a series of parallel physiological, cellular, and molecular events^6^. Plant responses to drought are complex, involving adaptive changes and/or deleterious effects^7^, and the various responses are modulated by the plant species as well as the intensity, duration, and rate of progression of the imposed stress^8^. Drought stress has profound effects on general plant physiology, i.e., both the gas exchange and water status depend on the rapidity, severity and duration of the drought event. The earliest response to a leaf water deficit is stomatal closure to prevent desiccation^9^,^10^, which occurs before any change in leaf water potential (LWP) and/or relative water content (RWC)^11^,^12^. It is now well established that there is drought-induced root-to-leaf signaling, such as that involving abscisic acid (ABA), which reaches the leaves through the transpiration stream and induces the closure of stomata^11^. The photosynthesis rate (*A*) is subsequently affected by the internal water deficiency, so *A* is unavoidably reduced due to decreased CO_2_ availability at the level of the chloroplast^13^. In recent years, stomatal closure has generally been accepted as the main determinant for decreased *A* under drought conditions^11^,^14^, and the primary role of the stomata might be to avoid damage from plant water deficits^15^. However, another possibility is that control of the transpiration rate (T_r_) by the stomata plays a role in maintaining leaf temperature under drought conditions^10^. The drought-stress-induced limitation on plant growth is mainly caused by reductions in carbon assimilation, which depends on the balance between *A* and respiration^16^. The response of *A* to drought stress has received considerable attention in the past, and efforts have been made to generalize the responses of photosynthetic parameters to drought in higher plants^17^,^18^,^19^. It has been emphasized that a high degree of co-regulation of stomatal conductance (g_s_) and *A* is usually observed^11^,^20^. Determining the effect of a given change in g_s_ on *A* and T_r_ can be fairly straightforward, but analyzing the role of stomata in the control of these changes is complicated because the changes in *A* and T_r_ that result from changes in g_s_ can themselves affect g_s_. Decreased *A* and T_r_ can affect the LWP, which then changes g_s_ through a feedback^10^. The g_s_ and *A* of leaves also decrease as water status declines^21^, but the precise relationship is dependent on several factors, including the study species, drought history and environmental conditions during drought^11^.

Plant responses to drought and the relationship between gas exchange and water status, which depend on drought intensity, metabolic CO_2_ assimilation and biological form, are clearly complex^22^. In past years, numerous works have focused on plant gas exchange and water status under drought, and all reports showed the decrease of gas exchange and water status trails in response to drought. However, the magnitudes of decrease differed greatly among the various studies. These differences were mainly the result of complex interactions between drought stress and the high variability of various plant types. Thus, it is difficult to compare the results of individual studies, and the responses of different plant types are unlikely to be effectively resolved by studies performed at individual sites. Therefore, to determine the central tendency and identify different patterns of plant response to drought, it is necessary to integrate results across studies. To this end, we conducted a meta-analysis based on global plant gas exchange and water status under drought and aimed to determine the changes in gas exchange (*A*, g_s_ and T_r_) and water status (leaf water potential and relative water content) of different types of plants and different drought intensities. The following issues were addressed in this study: (1) how gas exchange and water status respond to different drought intensities, photosynthetic pathways (C_3_ and C_4_) and growth forms (herbs, shrubs, trees and lianas) and the differences between C_3_ and C_4_ plants under the same drought intensities; (2) whether the relationships of both *A* and T_r_ with g_s_ shift in C_3_ and C_4_ plants, herbs, shrubs, trees and lianas during drought; and (3) the relationships between gas exchange and water status in various photosynthetic pathways and growth forms. Our results could improve the understanding of the response of plant gas exchange and water status to drought.

## Results

### Effects of drought on leaf gas exchange

**Figure 1:**
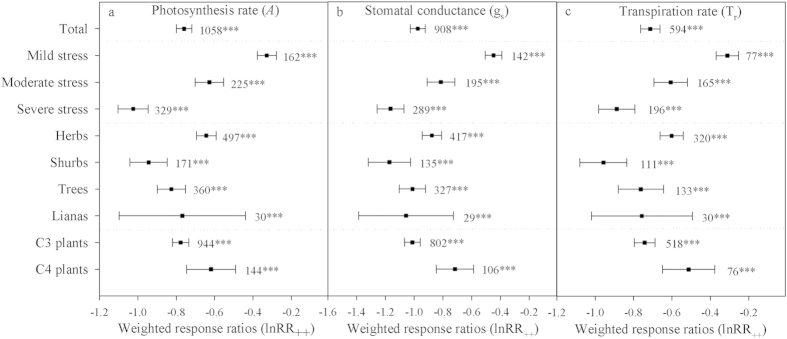
Weighted response ratios (lnRR_++_) of the plant photosynthesis rate (*A*, panel a), stomatal conductance (g_s_, panel b) and transpiration rate (T_r_, panel c) under different drought intensities (mild, moderate and severe) for different CO_2_-assimilation metabolic pathways (C_3_ and C_4_ plants) and in different growth forms (herbs, shrubs, trees and lianas). The numbers above the symbols specify the number of data points. The error bars indicate 95% CI. Symbols (***) after the number indicate statistical significance (P < 0.001).

Our results showed that the response ratios of *A*, g_s_ and T_r_ were −0.759 ± 0.020, −0.975 ± 0.026 and −0.713 ± 0.026 (P < 0.0001) (Fig. 1a–c), respectively, across all studies. When taking drought intensity into consideration, we found that the response ratio of *A* significantly decreased under drought compared to the control with values of −0.328 ± 0.025, −0.627 ± 0.038 and −1.024 ± 0.040 (P < 0.0001) under mild, moderate and severe drought, respectively. Furthermore, the response ratio of g_s_ also decreased significantly under drought compared to the control with values of −0.4479 ± 0.058, −0.8140 ± 0.097 and −1.1649 ± 0.093 (P < 0.0001) under mild, moderate and severe drought, respectively. When we divided the plant species into four growth forms (herbs, shrubs, trees and lianas), we found that herbs and shrubs showed the lowest and highest *A*, g_s_ and T_r_ response ratios (Fig. 1a–c), respectively. When the two photosynthetic pathways (the C_3_ and C_4_ cycles) were considered, we found that the response ratios of *A*, g_s_ and T_r_ in C_4_ plants (−0.619 ± 0.065, −0.716 ± 0.067 and −0.514 ± 0.069, respectively; P < 0.0001) were lower than those in C_3_ plants (−0.777 ± 0.021, −1.012 ± 0.028 and −0.742 ± 0.028, respectively; P < 0.0001) (Fig. 1a–c), indicating that C_4_ plants performed better in the context of drought.

### Effects of drought on leaf gas exchange in C_3_ and C_4_ plants under different intensities

**Figure 2:**
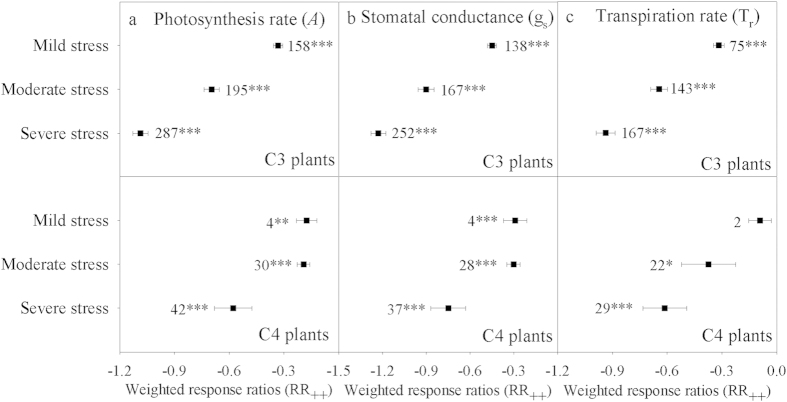
Weighted response ratios (lnRR_++_) of the plant photosynthesis rate (*A*, panel a), stomatal conductance (g_s_, panel b) and transpiration rate (T_r_, panel c) under different drought intensities (mild, moderate and severe) for different CO_2_-assimilation metabolic pathway (C_3_ and C_4_ plants). The numbers above the symbols specify the number of data points, and the error bars indicate 95% CI. The symbols (*, **, ***) after the number indicate statistical significance (P < 0.05, P < 0.01, P < 0.001, respectively).

Our results showed that the response ratios of *A*, g_s_ and T_r_ in C_3_ plants were higher than in C_4_ plants under the same drought intensities (Fig. 2a–c). The response ratios of *A*, g_s_ and T_r_ under severe drought stress were −1.086 ± 0.042, −1.228 ± 0.050 and −0.937 ± 0.052, respectively, in C3 plants and −0.578 ± 0.103, −0.750 ± 0.120 and −0.613 ± 0.120 in C_4_ plants. The response ratios of *A* and g_s_ did not differ under mild and moderate drought stress in C_4_ plants.

### Effects of drought on leaf water status

**Figure 3:**
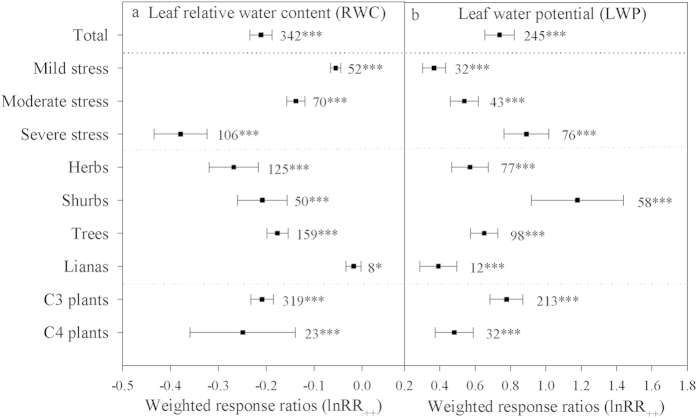
Weighted response ratios (lnRR_++_) of the relative water content (RWC) and leaf water potential (LWP) under different drought intensities (mild, moderate and severe) for different CO_2_-assimilation metabolic pathways (C_3_ and C_4_ plants) and in different growth forms (herbs, shrubs, trees and lianas). The numbers above the symbols specify the number of data points. The error bars indicate 95% CI. Symbols (*, **, ***) after the number indicate statistical significance (P < 0.05, P < 0.01, P < 0.001, respectively).

Leaf water potential (LWP) and relative water content (RWC) were the main indices used to reflect the water status of plants suffering from a drought, and the RWC and LWP response ratios across all studies were −0.211 ± 0.012 and 0.739 ± 0.042 (P < 0.0001) (Fig. 3a,b), respectively. The RWC and LWP response ratios differed according to drought intensity, resulting in values of −0.055 ± 0.006 and 0.367 ± 0.033, −0.138 ± 0.009 and 0.539 ± 0.040, and −0.379 ± 0.028 and 0.890 ± 0.065 (P < 0.0001) under mild, moderate and severe drought, respectively. The response ratios of RWC and LWP differed among growth forms with herbs exhibiting the highest RWC but a lower LWP and shrubs showing the highest LWP; lianas showed the lowest RWC and LWP values among the four plant types. C_4_ plants exhibited a higher RWC but a lower LWP.

### Relationships between stomatal conductance and both photosynthetic and transpiration rate

**Figure 4:**
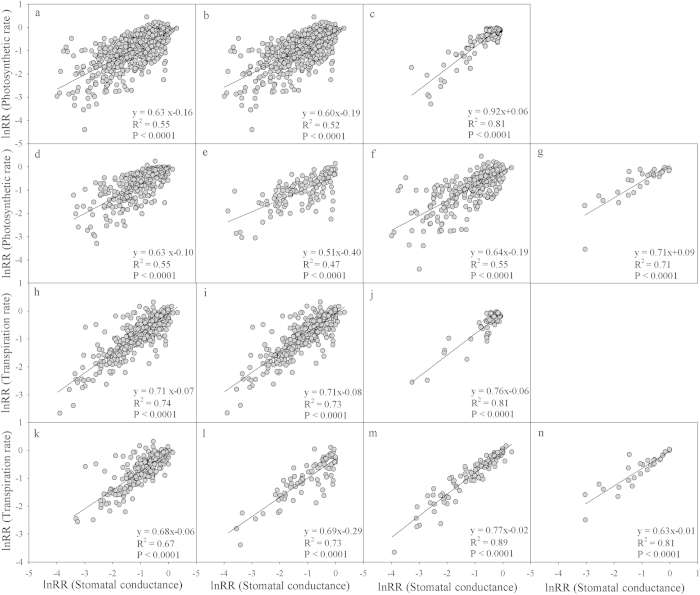
The relationships between the response ratios (lnRR) of the stomatal conductance (g_s_) with the photosynthesis rate (*A*) and transpiration rate (T_r_) in all plants (panels a,h), C_3_ plants (panel b,i), C_4_ plants (panel c,j), herbs (panels d,k), shrubs (panels e,l), trees (panels f,m) and lianas (panels g,n).

The stomata are the gates through which CO_2_ and water pass, and the results showed that the response ratio of stomatal conductance (lnRR(stomatal conductance)) correlated significantly with the response ratio of photosynthetic rate (lnRR(photosynthetic rate) = 0.63 lnRR(stomatal conductance) −0.16, P < 0.0001, R^2^ = 0.55) in all of the studies (Fig. 4a). When divided into the two photosynthetic pathways, the stomatal conductance response ratio also correlated significantly with the photosynthetic rate response ratio in C_3_ plants (lnRR(photosynthetic rate) = 0.60 lnRR(stomatal conductance) −0.19, P < 0.0001) (Fig. 4b) and C_4_ plants (lnRR(photosynthetic rate) = 0.92 lnRR(stomatal conductance) + 0.06, P < 0.0001) (Fig. 4c), which could explain 52% and 81% of the photosynthetic rate, respectively. The lnRR(stomatal conductance) also exhibited a significant correlation with lnRR(photosynthetic rate) in all four growth forms (P < 0.0001) (Fig. 4d–g), explaining 55%, 47%, 55% and 71% in herbs, shrubs, trees and lianas, respectively. The lnRR(stomatal conductance) correlated significantly with the transpiration rate response ratio (lnRR(transpiration rate)) (P < 0.0001) (Fig. 4h) and explained 74% of the lnRR(transpiration rate) in all of the studies; 73% and 81% of the lnRR(transpiration rate) in C_3_ and C_4_ plants, respectively (Fig. 4i,j); and 67%, 73%, 89% and 81% in herbs, shrubs, trees and lianas (Fig. 4k–n). The results also indicated that lnRR(stomatal conductance) contributes more to water exchange than to CO_2_ exchange (Fig. 4).

### Relationship between gas exchange and water status

**Figure 5:**
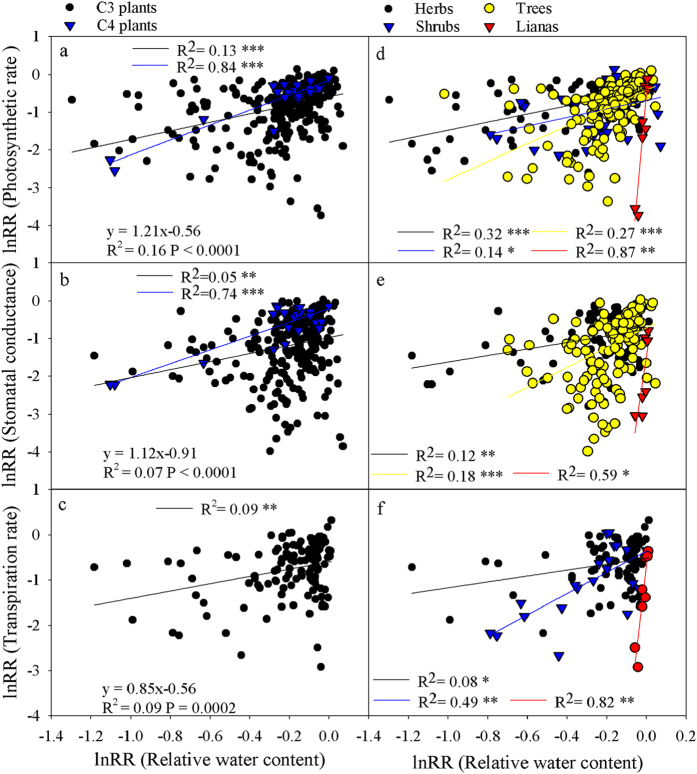
The relationship between the response ratios (lnRR) of the leaf relative water content (RWC) and gas exchange in all plants. The regression equation represents the correlation across all studies, and the black and blue lines represent the correlations of the C_3_ and C_4_ plants (panel **a**–**c**), respectively; the black, blue, yellow and red lines represent the correlations of the herbs, shrubs, trees and lianas (panels **d**–**f**), respectively.

**Figure 6:**
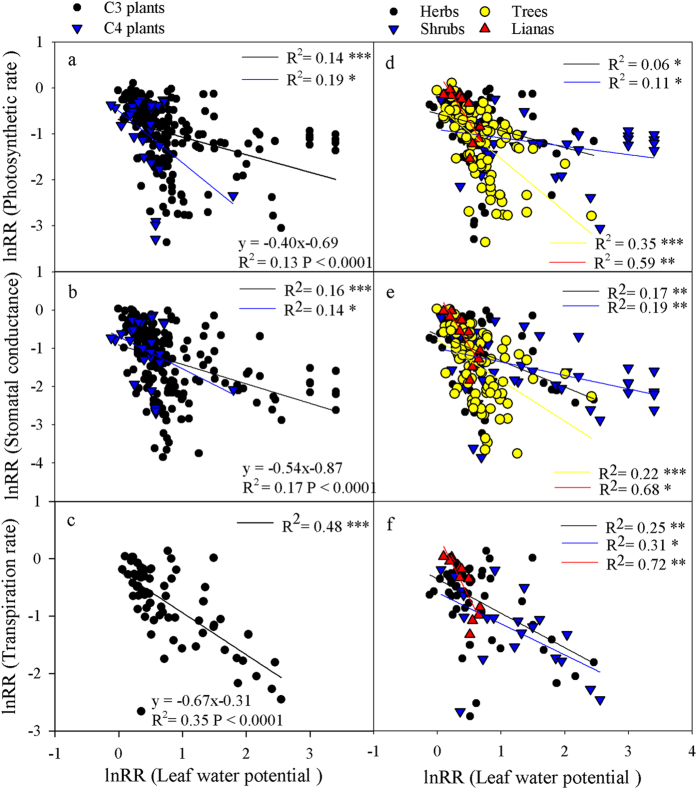
The relationship between the response ratios (lnRR) of the leaf water potential (LWP) and gas exchange in all plants. The regression equation represents the correlation across all studies, and the black and blue lines represent the correlation of the C_3_ and C_4_ plants (panels **a**–**c**), respectively; the black, blue, yellow and red lines represent the correlations of the herbs, shrubs, trees and lianas (panels **d**–**f**), respectively.

The results showed that the response ratios of *A*, g_s_ and T_r_ (P < 0.001) were significantly correlated with water status, including RWC and LWP across all studies (Figs 5 and 6). C_3_ plants showed a significant correlation between the response ratios of RWC and LWP and *A*, g_s_ and T_r_ (Figs 5a–c and 6a–c), but there were no correlations between the RWC and LWP response ratios and T_r_ in C_4_ plants. The RWC response ratio contributed more substantially to the *A* and g_s_ response ratios in C_4_ than in C_3_ plants. The response ratio of *A* showed a significant correlation with the response ratios of RWC and LWP in all four growth forms (Figs 5d and 6d) and a significant correlation between the g_s_ response ratio and LWP in all four growth forms (Fig. 6e) but no significant correlation between the response ratios of RWC and g_s_ in shrubs (Fig. 5e). Finally, no correlation was found between the T_r_ response ratio and RWC or LWP in trees (Figs 5f and 6f).

## Discussion

In this study, we conducted a meta-analysis of global gas exchange and water status data, and we analyzed the response ratios of these parameters under different drought intensities (mild, moderate and severe drought) and different plant growth forms (herbs, shrubs, trees and lianas) and photosynthetic pathways (C_3_ and C_4_). We also analyzed the response ratios of C_3_ and C_4_ plants under different drought intensities (Figs 1, 2, 3) as well as the relationship between gas exchange and water status in the different plant types and photosynthetic pathways (Figs 4, 5, 6). The stomata provide a means for controlling water loss from plants while allowing photosynthesis, so they play a fundamental role in determining plant transpiration and assimilation. We found that the g_s_ response ratio was greater than those of *A*, T_r_, RWC and LWP under different drought intensities, plant growth forms and photosynthetic pathways, and the decrease in lng_s_ could explain 55% of the change in ln*A* and 74% in lnT_r_ across all studies, respectively, which indicates that g_s_ plays an important role in controlling photosynthesis and transpiration rates. The g_s_ response was greater than the decrease in gas exchange, RWC and/or LWP, which is consistent with the results of Gollan *et al*.^18^, Chaves *et al*.^7^ and Jensen *et al*.^19^. Stomata are the gates through which carbon and water exchange occurs, and stomatal closure helps reduce water loss from transpiration, which could be regulated by signals from the roots in drying soil^23^,^24^. Although a high degree of g_s_ and *A* co-regulation was usually observed^11^,^20^, the decrease in *A* was smaller than that in g_s_ because of metabolic adjustments by the plants^11^,^21^.

The stress tolerance of different growth forms is an important factor that may affect the way plants respond to drought. In this study, there were no tolerant or sensitive plant genotypes, and all of them survived the imposed stress. Among the four growth forms, we observed that the gas exchange response ratio was smallest in herbs, indicating that their ability to adapt to drought conditions through regulation is relatively low and that a higher T_r_ in herbs could accelerate plant death in the context of drought. Shrubs and trees exhibited more rapid responses to drought and might have enhanced drought resistance due to their lower T_r_ and large roots. Gas exchange in C_4_ plants was less influenced by drought, and the C_4_ plants showed a smaller response than C_3_ plants under the same drought conditions (Fig. 3). This is consistent with reports that C_4_ plants have some advantages under drought conditions compared with C_3_ plants^25^.

It is generally accepted that the accurate measurement of plant water status is critical in experiments investigating the effects of drought and that such measurements must be considered when defining the experimental conditions in terms of both the treatments applied and the effects on the plants. Leaf water status depends on the soil water deficit, which can be regarded as a stressor^26^. In the study, the water status of plants showed a slight decrease under mild drought, followed by a large decrease under severe stress in this study, which is consistent with the results of Galmés *et al*.^17^. RWC, as the metabolically available water, could reflect the metabolic activity in plant tissues, and it declines with continuing drought. LWP, which could reflect the water transport, also declines with drought; thus, both RWC and LWP could as indicators for plants under drought. In this study, we found that the RWC response ratio was smaller than the LWP response ratio, indicating that LWP was more sensitive than RWC. This finding establishes LWP as an earlier indicator of drought than RWC, which differs from the conclusion of Sinclair and Ludlow^27^, who proposed that RWC was a better indicator. The lianas used in this meta-analysis come from four studies, and all the plant material was *Vitis vinifera* L. We found that the response ratio of water status in the lianas was the smallest among the growth forms, which may be due to the near-isohydric behavior of *Vitis vinifera*^28^,^29^,^30^. There were no significant differences in the response ratios of RWC in C_3_ (based on 152 papers and 139 species) and C_4_ (based on 21 papers and 13 species) plants, but the LWP response ratio in C_3_ plants was higher than that in C_4_ plants. This may be due to a difference in drought resistance between C_3_ and C_4_ plants^25^, which requires further investigation.

The stomata occupy a central position in the pathways for both water loss from plants and CO_2_ exchange. The debate regarding the main determinant of decreased *A* under drought has been ongoing since the publication of studies of the effects of drought on *A*^31^,^32^,^33^, which generally conclude that stomatal closure is the main determinant^11^,^14^. Very few studies have directly examined how the relationship between *A* and g_s_ is affected by drought in different plant growth forms. Stomata often close in response to drought before any change occurs in LWP and/or RWC^12^. The regulation of g_s_ is related to species and genotype, making it difficult to define a pattern of photosynthetic responses to drought. Furthermore, a high degree of *A* and g_s_ co-regulation is usually observed^11^,^20^; the decrease in g_s_ could explain 55% of the decline in *A* in all of the studies, indicating that, consistent with Cornic and Massacci^14^ and Medrano *et al*.^11^, the decrease in g_s_ is primarily responsible for the decline in *A* under drought conditions. Besides, the non-stomatal limitation was also responsible for the decline in *A*^11^,^31^, such as the decrease of mesophyll conductance, which was an important limiting factor in photosynthesis. However, due to the lack of mesophyll conductance data in our dataset, or the literature did not meet the other criteria for inclusion, the contribution of CO_2_ diffusion within the leaf could not be accurately obtained due to the limited data on mesophyll conductance, so this parameter was not included in this manuscript despite its importance. In C_4_ plants, the decrease in g_s_ explained 81% of the decline in *A*, suggesting that the decrease in g_s_ played a more important role in the decline in *A* under drought in the C_4_ plants; this finding is consistent with the findings of Da Silva and Arrabaca^34^, Ripley *et al*.^35^, Ghannoum *et al*.^36^ and Ghannoum^37^, who reported that the *A* in C_4_ plants under drought was mainly limited by the decline in g_s_ caused by stomatal closure. Moreover, we found that the growing temperature also influenced the decline in *A* (Supplementary information, Figure S1), indicating that warming temperatures may strengthen the severity of the effects of drought on plants.

In general, we know that T_r_ depends on g_s_, the air saturation deficit, temperature, wind speed, and other factors^38^. Rising temperatures could increase the T_r_ of plants (Figure S1), which could accelerate the loss of soil water. Although the role of stomata in the control of transpiration has been the subject of debate for many years, the role of stomata in controlling transpiration can be analogously defined as the relative change in T_r_ for a given relative change in g_s_^10^. Across all studies, we found that a decrease in g_s_ could explain 74% of the variance in T_r_, which is higher than that of *A*, indicating that maintaining plant water status may the most important function under drought stress. This finding was consistent with Cowan^39^,^40^, Parkhurst and Loucks^41^ and Jones^10^, who suggested that stomata operate in a manner that minimizes water loss relative to the ratio of CO_2_ uptake to soil moisture decrease. We also found that the decrease of g_s_ could explain the decline of T_r_ in C_4_ plants more than in C_3_ plants and that it was higher in trees than in herbs, shrubs and lianas, indicating that decrease of g_s_ caused by drought is likely primarily responsible for the decline of T_r_ in C_4_ plants and trees.

Gas exchange is known to be closely related to the status of leaf water, which could be considered to be an indicator of stress under drought conditions^26^. In this study, we found that gas exchange had a close relationship to leaf water status, as previous works reported that the *A* in plants decreased as the RWC and LWP decreased^14^,^21^,^42^. We also found that the decrease of RWC in C_4_ plants caused by drought was a major reason for the decrease of *A*. Additionally, *A* showed a faster decrease with the decline of leaf water status in C_4_ than C_3_ plants. Moreover, we also found that a decrease in leaf water status caused by drought could explain the decreased *A* to greatest extent in the lianas among the four plant types. In either case, plant water status had a significant relationship with g_s_^10^, and the results supported the notion that leaf water status influences the stomatal response under drought. In all of the studies, leaf water status showed a significant relationship with the g_s_, and g_s_ showed a faster decrease with the decline in leaf water status in C_4_ plants and lianas. The change of leaf water status also showed a significant relationship with T_r_. The absence of a strong relationship between water status and gas exchange indicated that other factors are involved in regulating gas exchange, such as the air saturation deficit, temperature, and wind speed^38^.

Our meta-analysis was based on the global scale and focused on the response of the gas exchange and water status to drought and the relationship between these two factors. Based on our data set, we concluded that drought should decrease the gas exchange and water status slightly under mild drought and substantially under severe stress. The g_s_ showed a larger decrease than other physiological traits, and the gas exchange declined the most in shrubs, compared with herbs, trees and lianas. Gas exchange also showed a more substantial decrease in C_3_ plants than in C_4_ plants. Moreover, the results showed that the decrease in g_s_ under drought conditions primarily reduced T_r_. Additionally, the decrease in g_s_ could explain 55% of the decrease in *A* and 74% of the decrease in T_r_ under drought, and it plays a relatively important role in the decrease in *A* in C_4_ plants and lianas. The gas exchange also showed a close relationship with the leaf water status, as RWC was less sensitive than LWP, and gas exchange showed a faster decrease in C4 plants and lianas as the water status decreased.

## Methods

### Data preparation

Peer-reviewed journal articles were searched using the Web of Science and the online databases of the Chinese Academy of Sciences with the following search term combinations: drought/water stress and photosynthesis/gas exchange. To avoid bias in the selection of publications, the studies were chosen based on the following criteria: (1) the experiments were conducted using at least two datasets (control and treatment) and included drought intensity, photosynthetic pathway (C_3_ and C_4_) and growth forms (herbs, shrubs, trees and lianas); (2) only experiments conducted under controlled conditions were included, and studies were excluded when the study plant was described as having both tolerant or sensitive genotypes and was not subject to drought-related mortality; and (3) the means, standard deviations/errors and sample sizes of the variables in the control and treatment groups could be directly extracted from the context, tables or digitized graphs. In addition, plant species, photosynthetic pathways (C_3_ or C_4_ plants), growth forms (herbs, shrubs, trees or lianas), drought intensity (mild, moderate or severe) and relative soil water content (RSWC) were recorded directly from the papers, and when the drought intensity was not provided by the study, it was grouped into one of three categories according to the RSWC: mild drought (55%<RSWC<70%), moderate drought (40%<RSWC<55%) and severe drought (RSWC<40%).

In total, 167 published papers involving 152 plant species (not include tolerant or sensitive genotypes) and reporting drought and/or water stress studies that satisfied our selection criteria for the meta-analysis were selected from more than 5,000 published papers (Supplementary information and Dataset), including 1,058 observations of *A*, 908 observations of g_s_, 594 observations of T_r_, 342 observations of RWC and 245 observations of LWP. All original data were extracted from the text, tables, figures and appendices of the publications.

When data were presented graphically, numerical data were obtained using Get–Data Graph Digitizer (ver. 2.20, Russian Federation). To test differences in the responses of plant gas exchange and water status to drought, three drought intensities, mild stress (55 plant species, 47 papers), moderate stress (69 plant species, 69 papers) and severe stress (85 plant species, 78 papers); two photosynthetic pathways, C_3_ (139 plant species, 152 papers) and C_4_ (13 plant species, 21 papers); and four growth forms, herbs (62 plant species, 87 papers), shrubs (33 plant species, 30 papers), trees (53 plant species, 51 papers) and lianas (4 plant species, 4 papers) were included.

### Analysis

We followed the methods of Hedges *et al*.^43^ to evaluate the responses of gas exchange and water status to drought. A response ratio (lnRR, the natural log of the ratio of the mean value of a variable of interest in the drought treatment to that in the control) was used to represent the magnitude of the effects of drought as follows:


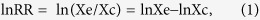


where Xe and Xc are the response values of each individual observation in the treatment and control, respectively. Because the LWP is a negative value, when calculating the lnRR of LWP, we used the absolute value of LWP. The corresponding sampling variance for each lnRR was calculated according to Eq. 2:


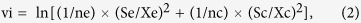


where ne, nc, Se, Sc, Xe and Xc are the sample sizes, standard deviations and mean response values in the experimental and control groups, respectively. The reciprocal of its variance (*W* = 1/vi) was considered as the weight of each lnRR. The mean weighted response ratio (RR_++_) was calculated from lnRR of individual pairwise comparisons between the treatment and control, lnRR_ij_ (*i* = *1, 2,…, m*; *j* = *1, 2,…, k*), as below:


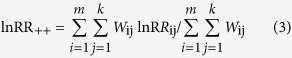


here, *m* is the number of groups (e.g., plant types), and *k* is the number of comparisons in the *i*th group.

The meta-analyses were performed using the R software package (version 3.1.1)^44^. The natural logs of the RRs for the individual and combined treatments were determined by specifying the studies as a random factor in the model in the “metafor” package. The effects of drought on gas exchange and water status were considered significant if the 95% confidence intervals (CIs) of lnRR did not overlap with zero. To compare the responses of gas exchange and water status to drought of different photosynthetic pathways (C_3_ and C_4_) and different growth forms (herbs, shrubs, trees and lianas) with the control, we tested whether the interactions between multiple treatments were significant by using the “rma.uni models” in the “metafor” package with treatments as the categorical variables. Regression analysis was conducted to detect relationships between the lnRR of gas exchange and water status under drought in the two photosynthetic pathways (C_3_ and C_4_) and four growth forms.

## Additional Information

**How to cite this article**: Yan, W. *et al*. A meta-analysis of leaf gas exchange and water status responses to drought. *Sci. Rep*. **6**, 20917; doi: 10.1038/srep20917 (2016).

## Supplementary Material

Supplementary Information

Supplementary Dataset
