# Supplementary material for: A meta-analysis of leaf gas exchange and water status responses to drought
Source: Sci Rep. 2016 Feb 12;6:20917. doi: 10.1038/srep20917 (PMC4751433; doi:10.1038/srep20917)
Supplement: Supplementary Information [file srep20917-s1.doc]

**Title page**

**Title:** A meta-analysis of leaf gas exchange and water status responses to drought

**Running title:** Meta-analysis of plants responses to drought

**Authors’ names:** Weiming Yan, Yangquanwei Zhong, Zhouping Shangguan*

**Authors’ institution:**

State Key Laboratory of Soil Erosion and Dryland Farming on the Loess Plateau, Northwest A&F University, Yangling, Shaanxi 712100, P.R. China

***Corresponding author:** Prof. Dr. Zhouping Shangguan

**Address:** Xinong Rd. 26, Institute of Soil and Water Conservation, Yangling, Shaanxi, 712100, P.R. China

Phone: ++86-29-87019107

Fax: ++86-29-87012210

E-mail: shangguan@ms.iswc.ac.cn

**Supporting information 1**

**A list of 167 papers from which the data were extracted for this meta-analysis.**

1. Ren L*, et al.* Varied morphological and physiological responses to drought stress among four tea Chrysanthemum cultivars. *Acta Ecologica Sinica* **35**, 01-12 (2015).

2. Zhang X, Gao J, Du W, Zhang R, Xue J. Effects of Drought Stress on Photosynthetic Characteristics of Maize Hybrids at Seedling Stage. *Acta Agronomica Sinica* **41**, 154-159 (2015).

3. Li S, Qin D, Wei Z, Qin W, Nie Z. Research on the photosynthesis of Manglietia tenuipesa under drought stress. *Northern Horticulture* **03**, 72-76 (2015).

4. Zhao Q, Pan J, Cao B, Song L. Effects of elevated temperature and drought stress on photosynthesis of Lycium barbarum. *Acta Ecologica Sinica* **35**, 01-10 (2015).

5. Wang Z*, et al.* Effects of Different Drought Stress on Photosynthesis and Activity of Photosystem II in Leaves of Amur Grape (Vitis amurensis). *Plant Physiology Journal* **50**, 1171-1176 (2014).

6. Ma S, Bai X, Qi L, Zhu J, Zhang Z. Changes in the growth and photosynthesis of cotton seedlings under progressive drought after saltwater irrigation. *Chinese Journal of Applied Ecology* **25**, 3521-3526 (2014).

7. Ding L, Li Y, Li Y, Shen Q, Guo S. Effects of drought stress on photosynthesis and water status of rice leaves. *Chinese Journal of Rice Science* **28**, 65-70 (2014).

8. Zhou Y*, et al.* Effects of Drought Stress on Photosynthetic Characteristics and Endogenous Hormone ABA and CTK Contents in Green-Stayed Sorghum. *Scientia Agricultura Sinica* **47**, 655-663 (2014).

9. Han B, Li Z, Guo H, Zhang J. Studies on Seedling Photosynthetic Characteristics of Five Tree Species under Drought Stress. *For Res* **27**, 092-098 (2014).

10. Jing D. Response of Photosynthetic Characteristics and Antioxidant Enzyme Activities in Poplar Seedlings to Drought Stress. *Journal of Nuclear Agricultural Sciences* **28**, 0532-0539 (2014).

11. Zhang J, Zhang Q, Sun G, He Q, Li X, Liu H. Effects of drought stress and re-watering on growth and photosynthesis of Hosta. *Acta Prataculturae Sinica* **23**, 167-176 (2014).

12. Yang Z, Zhao Y, Yu S, Xu L, Liu Z, Fan Y. The variations of physiological and root morphological characteristics of Dalbergia odorifera seedlings under drought stress. *China Forestry Science and Technology* **28**, 63-66 (2014).

13. Dang X*, et al.* Effects of Drought Stress on Anatomical Structure of Leave and Physiological Characteristics in Three Atriplex L. Seedling. *Acta Bot Boreali-Occidential Sinica* **34**, 0976-0987 (2014).

14. Wu J, Xu J, Zhang J, Wang L. Photosynthetic physiology response of two Potentilla species under drought stress. *Pratacultural Science* **31**, 1330-1335 (2014).

15. Zhong P, Li Y, Su S. The responses of photosynthetic and chlorophyII fluorescence to water stress in three provenances of Calligomum mongoicum. *Journal of Desert Research* **34**, 1301-1306 (2014).

16. Ye B, Wu Y, Shao W, Yang J. Effects of combined stress of elevated temperature and drought and of re-watering on the photosynthetic characteristics and chlorophyll fluorescence parameters of Broussonetia papyrifera seedlings. *Chinese Journal of Ecology* **33**, 2343-2349 (2014).

17. Yang W, Gu M, Kou J, Lei Z, Cheng J. Effect of drought and rewatering on the photosynthesis and chlorophyII fluorescence of Coronilla varia. *Acta Agrestia Sicica* **21**, 1130-1135 (2013).

18. Sun W, Sun Z, Wu Q, Liu H, Chen M, Chen Z. Effects of Elevated CO2 Concentration on Drought Tolerance in Transgenic Tomato Plants under Drought Stress. *Plant Physiology Journal* **49**, 1393-1399 (2013).

19. Liu F, Shen S, Li Y, Li L, Zou X. Effect of water stress on maize photosynthetic characteristics in different growth stages. *Journal of the Meteorological Sciences* **33**, 378-383 (2013).

20. Wang H, Zhang Y, Zhang S. Effect of Irrigation and Nitrogen Supply on Photosynthetic Characters and Yield of Broomcorn Millet. *Chinese Agricultural Science Bulletin* **29**, 259-267 (2013).

21. Guo S, Zhang M, Deng C, Na H, Wang X, Ye N. Effect of Drought Stress on Photosynthetic Characteristics and Ultrastructure of Mesophyll Cells in Three Cultivars of Jaminum sambac. *Journal of Tropical and Subtropical Botany* **21**, 123-129 (2013).

22. Pang J, Zhang F, Hao L, Yang Z, Zhao P. Effect of drought stress on anatomical structure and photosynthesis of Pugionium cornutum (L.) Gaertn. leaves in seedling. *Ecology and Environmental Sciences* **22**, 575-581 (2013).

23. Cai X, Chen X, Liu C, Zhang S, Fan T. Effect of drought stress with exogenous Ca2+ on relative water content and the characteristics of photosynthesis of Koelreuteria paniculata. *Northern Horticulture* **10**, 58-62 (2013).

24. Xue H, Zhang Y, Liu L, Sun H, Li C. Responses of Spectral Reflectance, Photosynthesis and Chlorophyll Fluorescence in Cotton During Drought Stress and Rewatering. *Scientia Agricultura Sinica* **46**, 2386-2393 (2013).

25. Liu J, Yan Q, Liao X, Yan G, Chi X, Li P. Effect of drought stress on photosynthesis of Drepanostachyum luodianense. *Guizhou Agricultural Sciences* **41**, 65-69 (2013).

26. Ji Y*, et al.* Effects of drought stress on the root growth and photosynthetic characters of Dactylis glomerata seedlings. *The journal of applied ecology* **24**, 2763-2769 (2013).

27. Hu W, Kang J, Liu Y, Chen X, Dong A. The Effect of Drought Stress on Photosynthetic Physiological Characteristics of the Different Tobacco Varieties. *Chinese Tobacco Science* **34**, 69-73 (2013).

28. Ma F, Li D, Cai J, Jiang D, Cao W, Dai T. Responses of wheat seedlings root growth and leaf photosynthesis to drought stress. *The journal of applied ecology* **23**, 724-730 (2012).

29. Li Q, Wang M, Wang W, Zhang R, Yue M. Response of photosynthetic characteristics of Psathyrostachys huashanica Keng to drought stress. *Acta Ecologica Sinica* **32**, 4278-4284 (2012).

30. Jin Y, Li D, Chen X, Zhang L. Physiological Response of Sapium sebiferum Seedlings from Different Provenances to Drought Stress [J]. *Acta Bot Boreali-Occidential Sinica* **32**, 1395-1402 (2012).

31. Zhang X, Jia Z, Zhu C, Zhang P, Kang L. Effect of water stress on photosynthesis and biomass of soybean during its pod filling stage. *Agricultural Research in the Arid Areas* **30**, 097-104 (2012).

32. Yang Y, Zhang Y, Peng F. Effects of drought stress on photosynthetic characteristics in Toona sinensis seedlings from different provenances. *Journal of Beijing Forestry University* **33**, 44-48 (2011).

33. Wang N, Hu Z, Shen Y. Photosynthetic characteristics of Davidia involucrata Baill. seedlings under soil drought stress. *Acta Bot Boreali-Occidential Sinica* **31**, 101-108 (2011).

34. Liu C, Liu Y, Guo K. Ecophysiological adaptations to drought stress of seedlings of four plant species with different growth forms in karst habitats. *Chinese Journal of Plant Ecology* **35**, 1070-1082 (2011).

35. Wang H, Liu L, Zhou D. Study on drought resistance of 4 Medicago L. varieties. *Acta Agrestia Sinica* **18**, 205-211 (2010).

36. Sun P, Duan X. Effects of Drought Stress on Soluble Sugars and Photosynthetic Characteristics of Catharanthus roseus Seedlings. *Journal of Northeast Forestry University* **38**, 054-056 (2010).

37. Wang Y*, et al.* Effects of drought stress on growth, photosynthetic physiological features and stomata characters of cucumber seedlings [J]. *J China Agric Univ* **15**, 12-18 (2010).

38. Kong D, Yu H, Li Y, Tian Y. Effect of drought stress on photosynthesis and physiologica characteristics of Chrysanthemum morifolium [J]. *Journal of Northwest A & F University (Natural Science Edition)* **38**, 103-108 (2010).

39. Li Z, Luo Q, Wu W, Han L. The effects of Drought Stress on Photosynthetic and Chlorophyll Fluorescence Characteristics of Populus euphratica and P. pruinosa. *Arid Zone Research* **26**, 45-52 (2009).

40. Ai J, Jin S. Effects of drought stress on photosynthetic characteristics in Hemsleya zhejiangensis [J]. *Chinese Traditional and Herbal Drugs* **39**, 1076-1078 (2008).

41. Wei L, Cui S. The Effect of Soil Drought Stress on Photosynthetic Character of Prunus armeniaca. *Acta Agriculturae Boreali-Sinica* **23**, 194-197 (2008).

42. Chu J, Meng P, Zhang J, Gao J. Effects of Soil Water Stress on the Photosynthesis Characteristics and Chlorophyll Fluorescence Parameters of Cerasus humilis Seedling. *For Res* **21**, 295-300 (2008).

43. Li W, Zhang S, Shan L. Effect of water stress on characteristics of root water uptake and photosynthesis in alfalfa seedlings. *Acta Agrestia Sinica* **15**, 206-211 (2007).

44. Li Y, Zhai M, Li Y, Yan S, Yang Q. The Changes of Photosynthesis and Anti-oxidase Activities in Paeonia suffruficosa Leaves under Draught Stress. *Journal of Henan Agricultural Sciences* **5**, 91-93 (2007).

45. Fu S, He X, Chen W, Wu H. Effect of NO on photosynthesis in different drought-enduring poplars. *Journal of Liaoning Technical University* **26**, 308-311 (2007).

46. Song L, Cai T, Yu X. Influence of water stress on the photosynthetic and physiological characteristic of Acanthopanax senticosus seedings. *Science of Soil and Water Conservation* **5**, 91-95 (2007).

47. Ke S. Photosynthetic Characteristics of Sinocalycanthus chinensis Leaves under Drought Stress. *Acta Bot Boreali-Occidential Sinica* **27**, 1209-1215 (2007).

48. Han R, Lu X, Gao G, Yang X. Photosynthetic physiological response of alfalfa (Medicago sativa) to drought stress. *Acta Ecologica Sinica* **27**, 5229-5237 (2007).

49. Liu D, Chen X. Characteristics of water consumption and utilization of Populus alba X berolinensis under water stress. *hinese Journal of Ecology* **25**, 290-294 (2006).

50. Ren L, Wang Y, Weng B, Fang J, Ying Z, Huan Y. The Effect of Water Stress on the Water Content and Photosynthesis of Leaves of Chamaecrista rotundifolia. *Journal of Xiamen University (Natural Science) S* **44**, 28-31 (2005).

51. Jing M, Cao F, Wang G, Hao M. The effects of soil water contents on photosynthetic characteristics of Ginkgo. *Journal of Nanjing Forestry University* **29**, 83-86 (2004).

52. Ren H, Chen X, Wu D. Effects of elevated CO2 on photosynthesis and antioxidative ability of broad bean plants grown under drought condition. *Acta Agronomica Sinica* **27**, 729-736 (2000).

53. Ren H, Chen X, Sun G, Wang Y. Response of wheat seedlings with different drought resistance to water deficiency and NaCl stresses. *The journal of applied ecology* **11**, 718-722 (2000).

54. Chen X, Wu D, Wang G, Ren H. Effect of elevated CO2 concentration on photosynthesis and antioxidative enzyme activities of wheat plant grown under drought condition. *The journal of applied ecology* **11**, 881-884 (2000).

55. Miao Y, Zhu Z, Guo Q, Ma H, Zhu L. Alternate wetting and drying irrigation-mediated changes in the growth, photosynthesis and yield of the medicinal plant Tulipa edulis. *Industrial Crops and Products* **66**, 81-88 (2015).

56. Miranda‐Apodaca J, Pérez‐López U, Lacuesta M, Mena‐Petite A, Muñoz‐Rueda A. The type of competition modulates the ecophysiological response of grassland species to elevated CO2 and drought. *Plant Biol* **17**, 298-310 (2015).

57. Singh R, Pandey N, Kumar A, Shirke PA. Physiological performance and differential expression profiling of genes associated with drought tolerance in root tissue of four contrasting varieties of two Gossypium species. *Protoplasma*, 1-12 (2015).

58. Sicher R, Bunce J, Barnaby J, Bailey B. Water-deficiency effects on single leaf gas exchange and on C4 pathway enzymes of maize genotypes with differing abiotic stress tolerance. *Photosynthetica* **53**, 3-10 (2015).

59. Karimi S, Yadollahi A, Arzani K, Imani A, Aghaalikhani M. Gas-exchange response of almond genotypes to water stress. *Photosynthetica*, 1-6 (2015).

60. Shen X, Dong Z, Chen Y. Drought and UV-B radiation effect on photosynthesis and antioxidant parameters in soybean and maize. *Acta Physiologiae Plantarum* **37**, 1-8 (2015).

61. AbdElgawad H, Farfan-Vignolo ER, de Vos D, Asard H. Elevated CO 2 mitigates drought and temperature-induced oxidative stress differently in grasses and legumes. *Plant Sci* **231**, 1-10 (2015).

62. Silva EN, Silveira JA, Ribeiro RV, Vieira SA. Photoprotective function of energy dissipation by thermal processes and photorespiratory mechanisms in Jatropha curcas plants during different intensities of drought and after recovery. *Environ Exp Bot* **110**, 36-45 (2015).

63. Salazar-Parra C*, et al.* Carbon balance, partitioning and photosynthetic acclimation in fruit-bearing grapevine (Vitis vinifera L. cv. Tempranillo) grown under simulated climate change (elevated CO 2, elevated temperature and moderate drought) scenarios in temperature gradient greenhouses. *J Plant Physiol* **174**, 97-109 (2015).

64. Gan L, Wu X, Zhong Y. Exogenously Applied Nitric Oxide Enhances the Drought Tolerance in Hulless Barley. *Plant Production Science* **18**, 52-56 (2015).

65. Li J*, et al.* Rice leaf heterogeneity in chlorophyll fluorescence parameters under short-term osmotic stress. *Biol Plant* **59**, 187-192 (2015).

66. Mutava RN*, et al.* Understanding abiotic stress tolerance mechanisms in soybean: A comparative evaluation of soybean response to drought and flooding stress. *Plant Physiol Biochem* **86**, 109-120 (2015).

67. Mantovani D, Veste M, Freese D. Black locust (Robinia pseudoacacia L.) ecophysiological and morphological adaptations to drought and their consequence on biomass production and water-use efficiency. *New Zealand Journal of Forestry Science* **44**, 1-11 (2014).

68. Jedmowski C, Bayramov S, Brüggemann W. Comparative analysis of drought stress effects on photosynthesis of Eurasian and North African genotypes of wild barley. *Photosynthetica* **52**, 564-573 (2014).

69. Mantovani D*, et al.* Transpiration and biomass production of the bioenergy crop Giant Knotweed Igniscum under various supplies of water and nutrients. *Journal of Hydrology and Hydromechanics* **62**, 316-323 (2014).

70. Tian F, Jia T, Yu B. Physiological regulation of seed soaking with soybean isoflavones on drought tolerance of Glycine max and Glycine soja. *Plant Growth Regulation* **74**, 229-237 (2014).

71. Topbjerg HB*, et al.* Physiological factors affecting intrinsic water use efficiency of potato clones within a dihaploid mapping population under well-watered and drought-stressed conditions. *Scientia Horticulturae* **178**, 61-69 (2014).

72. Toscano S, Scuderi D, Giuffrida F, Romano D. Responses of Mediterranean ornamental shrubs to drought stress and recovery. *Scientia Horticulturae* **178**, 145-153 (2014).

73. Dani KGS, Jamie IM, Prentice IC, Atwell BJ. Increased ratio of electron transport to net assimilation rate supports elevated isoprenoid emission rate in eucalypts under drought. *Plant Physiol* **166**, 1059-1072 (2014).

74. Saglam A, Kadioglu A, Demiralay M, Terzi R. Leaf rolling reduces photosynthetic loss in maize under severe drought. *Acta Bot Croat* **73**, 315-323 (2014).

75. Ogbaga CC, Stepien P, Johnson GN. Sorghum (Sorghum bicolor) varieties adopt strongly contrasting strategies in response to drought. *Physiol Plant* **152**, 389-401 (2014).

76. Guo Q, Zhang W, Li H. Comparison of photosynthesis and antioxidative protection in Sophora moorcroftiana and Caragana maximovicziana under water stress. *Journal of Arid Land* **6**, 637-645 (2014).

77. Husen A, Iqbal M, Aref IM. Growth, water status, and leaf characteristics of Brassica carinata under drought and rehydration conditions. *Brazilian Journal of Botany* **37**, 217-227 (2014).

78. Liu P, Yin L, Deng X, Wang S, Tanaka K, Zhang S. Aquaporin-mediated increase in root hydraulic conductance is involved in silicon-induced improved root water uptake under osmotic stress in Sorghum bicolor L. *J Exp Bot*, eru220 (2014).

79. Yang Y, Tang M, Sulpice R, Chen H, Tian S, Ban Y. Arbuscular mycorrhizal fungi alter fractal dimension characteristics of Robinia pseudoacacia L. seedlings through regulating plant growth, leaf water status, photosynthesis, and nutrient concentration under drought stress. *J Plant Growth Regul* **33**, 612-625 (2014).

80. Yang W*, et al.* Severe water deficit-induced ethylene production decreases photosynthesis and photochemical efficiency in flag leaves of wheat. *Photosynthetica* **52**, 341-350 (2014).

81. Ma C, Wang Z, Zhang L, Sun M, Lin T. Photosynthetic responses of wheat (Triticum aestivum L.) to combined effects of drought and exogenous methyl jasmonate. *Photosynthetica* **52**, 377-385 (2014).

82. Oliveira MT, Medeiros CD, Frosi G, Santos MG. Different mechanisms drive the performance of native and invasive woody species in response to leaf phosphorus supply during periods of drought stress and recovery. *Plant Physiol Biochem* **82**, 66-75 (2014).

83. Song Y, Ci D, Tian M, Zhang D. Comparison of the physiological effects and transcriptome responses of Populus simonii under different abiotic stresses. *Plant Mol Biol* **86**, 139-156 (2014).

84. Minghua Luo, Yaojin Hu, JINGTIAN YANG, SU Z. Effects of drought stress on leaf gas exchange and chlorophyII fluorescence of salvjamaltiorrhiza. *Chinese Journal of Applied Ecology*, 619-623 (2010).

85. Ming-hua LYL. EFFECTS OF DROUGHT STRESS ON GAS EXCHANGE CHARACTERISTICS AND PROTECTIVE ENZYME ACTIVITIES IN TWO VARIETIES OF Salvia miltiorrhiza Bge. SEEDLINGS [J]. *Journal of Nuclear Agricultural Sciences* **2**, 034 (2011).

86. Haiyan Liu, Jiyue Li, Yan Zhao, Kankan H. Influence of drought stress on gas exchange and water use efficiency salix psammophila growing in five places *Arid Zone Research* **24**, 815-820 (2008).

87. Zivcak M, Kalaji HM, Shao H-B, Olsovska K, Brestic M. Photosynthetic proton and electron transport in wheat leaves under prolonged moderate drought stress. *J Photochem Photobiol B: Biol* **137**, 107-115 (2014).

88. Tattini M*, et al.* Isoprene production in transgenic tobacco alters isoprenoid, non‐structural carbohydrate and phenylpropanoid metabolism, and protects photosynthesis from drought stress. *Plant, cell & environment* **37**, 1950-1964 (2014).

89. Hoover D, Knapp A, Smith M. Contrasting sensitivities of two dominant C4 grasses to heat waves and drought. *Plant Ecol* **215**, 721-731 (2014).

90. Wu S, Liang D, Ma F. Leaf micromorphology and sugar may contribute to differences in drought tolerance for two apple cultivars. *Plant Physiol Biochem* **80**, 249-258 (2014).

91. Nogués I*, et al.* Physiological and antioxidant responses of Quercus ilex to drought in two different seasons. *Plant Biosystems-An International Journal Dealing with all Aspects of Plant Biology* **148**, 268-278 (2014).

92. Martorell S, DIAZ‐ESPEJO A, Medrano H, Ball MC, Choat B. Rapid hydraulic recovery in Eucalyptus pauciflora after drought: linkages between stem hydraulics and leaf gas exchange. *Plant, cell & environment* **37**, 617-626 (2014).

93. Mak M*, et al.* Leaf mesophyll K+, H+ and Ca 2+ fluxes are involved in drought-induced decrease in photosynthesis and stomatal closure in soybean. *Environ Exp Bot* **98**, 1-12 (2014).

94. Guha A, Reddy AR. Leaf gas exchange, water relations and photosystem-II functionality depict anisohydric behavior of drought-stressed mulberry (Morus indica, cv. V1) in the hot semi-arid steppe agroclimate of Southern India. *Flora-Morphology, Distribution, Functional Ecology of Plants* **209**, 142-152 (2014).

95. BARBOSA MAM*, et al.* Photosynthesis-Involvement in Modulation of Ascorbate and Glutathione in Euterpe oleracea Plants Exposed to Drought. *Notulae Botanicae Horti Agrobotanici Cluj-Napoca* **42**, 119-127 (2014).

96. Koller S, Holland V, Brüggemann W. Effects of drought stress on the evergreen Quercus ilex L., the deciduous Q. robur L. and their hybrid Q.× turneri Willd. *Photosynthetica* **51**, 574-582 (2013).

97. Ashraf M, Ali Q, Ashraf MA. Assessment of variation in drought tolerance using some key physiological criteria in potential wheat (Triticum aestivum L.) cultivars of different geographic origins. *Archives of Agronomy and Soil Science* **59**, 1503-1516 (2013).

98. Li D*, et al.* Effects of elevated CO 2 on the growth, seed yield, and water use efficiency of soybean (Glycine max (L.) Merr.) under drought stress. *Agric Water Manage* **129**, 105-112 (2013).

99. Sengupta D, Guha A, Reddy AR. Interdependence of plant water status with photosynthetic performance and root defense responses in Vigna radiata (L.) Wilczek under progressive drought stress and recovery. *J Photochem Photobiol B: Biol* **127**, 170-181 (2013).

100. Alexou M. Development-specific responses to drought stress in Aleppo pine (Pinus halepensis Mill.) seedlings. *Tree physiology*, tpt084 (2013).

101. Jiménez S*, et al.* Physiological, biochemical and molecular responses in four Prunus rootstocks submitted to drought stress. *Tree physiology*, tpt074 (2013).

102. Ranjbarfordoei A, Vandamme P, Samson R. Some ecophysiological characteristics of artà (Calligonum comosum Ľ Hérit) in response to drought stress. *Forest Science and Practice* **15**, 114-120 (2013).

103. Silva PE, Cavatte PC, Morais LE, Medina EF, DaMatta FM. The functional divergence of biomass partitioning, carbon gain and water use in Coffea canephora in response to the water supply: Implications for breeding aimed at improving drought tolerance. *Environ Exp Bot* **87**, 49-57 (2013).

104. Osório M, Osório J, Romano A. Photosynthesis, energy partitioning, and metabolic adjustments of the endangered Cistaceae species Tuberaria major under high temperature and drought. *Photosynthetica* **51**, 75-84 (2013).

105. Bae C-Y*, et al.* Physiological Responses of Calystegia soldanella under Drought Stress. *Journal of Ecology and Environment* **36**, 255-265 (2013).

106. Huang C-j*, et al.* Alteration in yield, gas exchange and chlorophyll synthesis of ramie to progressive drought stress. *Journal of Food, Agriculture & Environment* **11**, 302-305 (2013).

107. Feistler A, Habermann G. Assessing the role of vertical leaves within the photosynthetic function of Styrax camporum under drought conditions. *Photosynthetica* **50**, 613-622 (2012).

108. Greco M, Chiappetta A, Bruno L, Bitonti MB. In Posidonia oceanica cadmium induces changes in DNA methylation and chromatin patterning. *J Exp Bot*, err313 (2011).

109. Fang X, Turner N, Li F, Li W, Guo X. Caragana korshinskii seedlings maintain positive photosynthesis during short-term, severe drought stress. *Photosynthetica* **49**, 603-609 (2011).

110. Prasad P, Pisipati S, Momčilović I, Ristic Z. Independent and Combined Effects of High Temperature and Drought Stress During Grain Filling on Plant Yield and Chloroplast EF‐Tu Expression in Spring Wheat. *Journal of Agronomy and Crop Science* **197**, 430-441 (2011).

111. Xin ZL, Mei G, Li S, Li S, Liang Z. Growth, water status and photosynthesis in two maize (Zea mays L.) cultivars as affected by supplied nitrogen form and drought stress. *Pakistan J Bot* **43**, 1995-2001 (2011).

112. Ali Q, Ashraf M. Induction of drought tolerance in maize (Zea mays L.) due to exogenous application of trehalose: growth, photosynthesis, water relations and oxidative defence mechanism. *Journal of Agronomy and Crop Science* **197**, 258-271 (2011).

113. Ghaderi N, Talaie A, Ebadi A, Lessani H. The physiological response of three Iranian grape cultivars to progressive drought stress. *Journal of Agricultural Science and Technology* **13**, 601-609 (2011).

114. Liu C-C*, et al.* Comparative ecophysiological responses to drought of two shrub and four tree species from karst habitats of southwestern China. *Trees* **25**, 537-549 (2011).

115. Yang Y, Liu Q, Wang G. Physiological behaviors of Acer mono under drought and low light. *Russian Journal of Plant Physiology* **58**, 531-537 (2011).

116. Anjum S*, et al.* Gas exchange and chlorophyll synthesis of maize cultivars are enhanced by exogenously-applied glycinebetaine under drought conditions. *Plant Soil Environ* **57**, 326-331 (2011).

117. Vanaja M*, et al.* Response of C4 (maize) and C3 (sunflower) crop plants to drought stress and enhanced carbon dioxide concentration. *Plant, Soil and Environment* **57**, 207-215 (2011).

118. Hu W, Xiao Y, Zeng J, Hu X. Photosynthesis, respiration and antioxidant enzymes in pepper leaves under drought and heat stresses. *Biol Plant* **54**, 761-765 (2010).

119. Du N, Guo W, Zhang X, Wang R. Morphological and physiological responses of Vitex negundo L. var. heterophylla (Franch.) Rehd. to drought stress. *Acta physiologiae plantarum* **32**, 839-848 (2010).

120. Ditmarová Ľ, Kurjak D, Palmroth S, Kmeť J, Střelcová K. Physiological responses of Norway spruce (Picea abies) seedlings to drought stress. *Tree physiology*, tpp116 (2009).

121. Krouma A. Plant water relations and photosynthetic activity in three Tunisian chickpea (Cicer arietinum L.) genotypes subjected to drought. *Turk J Agric For* **34**, 257-264 (2010).

122. Silva EN, Ferreira-Silva SL, de Vasconcelos Fontenele A, Ribeiro RV, Viégas RA, Silveira JAG. Photosynthetic changes and protective mechanisms against oxidative damage subjected to isolated and combined drought and heat stresses in Jatropha curcas plants. *J Plant Physiol* **167**, 1157-1164 (2010).

123. Posch S, Bennett L. Photosynthesis, photochemistry and antioxidative defence in response to two drought severities and with re‐watering in Allocasuarina luehmannii. *Plant Biol* **11**, 83-93 (2009).

124. Vu JC, Allen LH. Growth at elevated CO 2 delays the adverse effects of drought stress on leaf photosynthesis of the C 4 sugarcane. *J Plant Physiol* **166**, 107-116 (2009).

125. de Magalhães Erismann N, Machado EC, Tucci MLSA. Photosynthetic limitation by CO2 diffusion in drought stressed orange leaves on three rootstocks. *Photosynthesis Res* **96**, 163-172 (2008).

126. Gomes FP, Oliva MA, Mielke MS, de Almeida A-AF, Leite HG, Aquino LA. Photosynthetic limitations in leaves of young Brazilian Green Dwarf coconut (Cocos nucifera L.‘nana’) palm under well-watered conditions or recovering from drought stress. *Environ Exp Bot* **62**, 195-204 (2008).

127. Gallé A, Feller U. Changes of photosynthetic traits in beech saplings (Fagus sylvatica) under severe drought stress and during recovery. *Physiol Plant* **131**, 412-421 (2007).

128. Poulos H, Goodale U, Berlyn G. Drought response of two Mexican oak species, Quercus laceyi and Q. sideroxyla (Fagaceae), in relation to elevational position. *Am J Bot* **94**, 809-818 (2007).

129. Robredo A*, et al.* Elevated CO 2 alleviates the impact of drought on barley improving water status by lowering stomatal conductance and delaying its effects on photosynthesis. *Environ Exp Bot* **59**, 252-263 (2007).

130. Germ M, Kreft I, Stibilj V, Urbanc-Berčič O. Combined effects of selenium and drought on photosynthesis and mitochondrial respiration in potato. *Plant Physiol Biochem* **45**, 162-167 (2007).

131. Ripley BS, Gilbert ME, Ibrahim DG, Osborne CP. Drought constraints on C4 photosynthesis: stomatal and metabolic limitations in C3 and C4 subspecies of Alloteropsis semialata. *J Exp Bot* **58**, 1351-1363 (2007).

132. Gallé A, Haldimann P, Feller U. Photosynthetic performance and water relations in young pubescent oak (Quercus pubescens) trees during drought stress and recovery. *New Phytol* **174**, 799-810 (2007).

133. Dias M, Brüggemann W. Differential inhibition of photosynthesis under drought stress in Flaveria species with different degrees of development of the C4 syndrome. *Photosynthetica* **45**, 75-84 (2007).

134. Praxedes SC, DaMatta FM, Loureiro ME, Ferrao MA, Cordeiro AT. Effects of long-term soil drought on photosynthesis and carbohydrate metabolism in mature robusta coffee (Coffea canephora Pierre var. kouillou) leaves. *Environ Exp Bot* **56**, 263-273 (2006).

135. Erice G, Irigoyen JJ, Pérez P, Martínez-Carrasco R, Sánchez-Díaz M. Effect of elevated CO 2, temperature and drought on dry matter partitioning and photosynthesis before and after cutting of nodulated alfalfa. *Plant Sci* **170**, 1059-1067 (2006).

136. Monti A, Brugnoli E, Scartazza A, Amaducci M. The effect of transient and continuous drought on yield, photosynthesis and carbon isotope discrimination in sugar beet (Beta vulgaris L.). *J Exp Bot* **57**, 1253-1262 (2006).

137. Yin C, Berninger F, Li C. Photosynthetic responses of Populus przewalski subjected to drought stress. *Photosynthetica* **44**, 62-68 (2006).

138. Duan B, Lu Y, Yin C, Junttila O, Li C. Physiological responses to drought and shade in two contrasting Picea asperata populations. *Physiol Plant* **124**, 476-484 (2005).

139. Aranda I, Castro L, Pardos M, Gil L, Pardos J. Effects of the interaction between drought and shade on water relations, gas exchange and morphological traits in cork oak (Quercus suber L.) seedlings. *For Ecol Manage* **210**, 117-129 (2005).

140. Ratnayaka H, Kincaid D. Gas exchange and leaf ultrastructure of Tinnevelly senna, under drought and nitrogen stress. *Crop Sci* **45**, 840-847 (2005).

141. Miyashita K, Tanakamaru S, Maitani T, Kimura K. Recovery responses of photosynthesis, transpiration, and stomatal conductance in kidney bean following drought stress. *Environ Exp Bot* **53**, 205-214 (2005).

142. Yin C, Peng Y, Zang R, Zhu Y, Li C. Adaptive responses of Populus kangdingensis to drought stress. *Physiol Plant* **123**, 445-451 (2005).

143. Tezara W, Marín O, Rengifo E, Martínez D, Herrera A. Photosynthesis and photoinhibition in two xerophytic shrubs during drought. *Photosynthetica* **43**, 37-45 (2005).

144. Egilla J, Davies Jr F, Boutton T. Drought stress influences leaf water content, photosynthesis, and water-use efficiency of Hibiscus rosa-sinensis at three potassium concentrations. *Photosynthetica* **43**, 135-140 (2005).

145. Lauriano J, Ramalho J, Lidon F, do Céu Matos M. Peanut photosynthesis under drought and re-watering. *Photosynthetica* **42**, 37-41 (2004).

146. Inoue T, Inanaga S, Sugimoto Y, An P, Eneji A. Effect of drought on ear and flag leaf photosynthesis of two wheat cultivars differing in drought resistance. *Photosynthetica* **42**, 559-565 (2004).

147. Inoue T, Inanaga S, Sugimoto Y, El Siddig K. Contribution of pre-anthesis assimilates and current photosynthesis to grain yield, and their relationships to drought resistance in wheat cultivars grown under different soil moisture. *Photosynthetica* **42**, 99-104 (2004).

148. Poulson ME, Donahue RA, Konvalinka J, Boeger MRT. Enhanced tolerance of photosynthesis to high-light and drought stress in Pseudotsuga menziesii seedlings grown in ultraviolet-B radiation. *Tree physiology* **22**, 829-838 (2002).

149. Clifford SC, Stronach IM, Black CR, Singleton‐Jones PR, Azam‐Ali SN, Crout NM. Effects of elevated CO2, drought and temperature on the water relations and gas exchange of groundnut (Arachis hypogaea) stands grown in controlled environment glasshouses. *Physiol Plant* **110**, 78-88 (2000).

150. Warren C, Bleby T, Adams M. Changes in gas exchange versus leaf solutes as a means to cope with summer drought in Eucalyptus marginata. *Oecologia* **154**, 1-10 (2007).

151. Guerfel M, Baccouri O, Boujnah D, Zarrouk M. Changes in lipid composition, water relations and gas exchange in leaves of two young ‘Chemlali’and ‘Chetoui’olive trees in response to water stress. *Plant Soil* **311**, 121-129 (2008).

152. Hura T, Hura K, Grzesiak M, Rzepka A. Effect of long-term drought stress on leaf gas exchange and fluorescence parameters in C3 and C4 plants. *Acta Physiologiae Plantarum* **29**, 103-113 (2007).

153. Ohashi Y, Nakayama N, Saneoka H, Fujita K. Effects of drought stress on photosynthetic gas exchange, chlorophyll fluorescence and stem diameter of soybean plants. *Biol Plant* **50**, 138-141 (2006).

154. Wall GW, Garcia RL, Wechsung F, Kimball BA. Elevated atmospheric CO 2 and drought effects on leaf gas exchange properties of barley. *Agriculture, ecosystems & environment* **144**, 390-404 (2011).

155. Elsheery NI, Cao K-F. Gas exchange, chlorophyll fluorescence, and osmotic adjustment in two mango cultivars under drought stress. *Acta Physiologiae Plantarum* **30**, 769-777 (2008).

156. Tsuji W, Ali M, Inanaga S, Sugimoto Y. Growth and gas exchange of three sorghum cultivars under drought stress. *Biol Plant* **46**, 583-587 (2003).

157. Ashraf M, Ashraf M, Khaliq A, Rha ES. Growth and leaf gas exchange characteristics in Dalbergia sissoo Roxb. and D. latifolia Roxb. under water deficit. *Photosynthetica* **42**, 157-160 (2004).

158. Mahouachi J. Changes in nutrient concentrations and leaf gas exchange parameters in banana plantlets under gradual soil moisture depletion. *Scientia Horticulturae* **120**, 460-466 (2009).

159. Silva Ed, Ribeiro R, Ferreira-Silva S, Viégas R, Silveira J. Comparative effects of salinity and water stress on photosynthesis, water relations and growth of Jatropha curcas plants. *J Arid Environ* **74**, 1130-1137 (2010).

160. Thameur A, Lachiheb B, Ferchichi A. Drought effect on growth, gas exchange and yield, in two strains of local barley Ardhaoui, under water deficit conditions in southern Tunisia. *J Environ Manage* **113**, 495-500 (2012).

161. Petridis A, Therios I, Samouris G, Koundouras S, Giannakoula A. Effect of water deficit on leaf phenolic composition, gas exchange, oxidative damage and antioxidant activity of four Greek olive (Olea europaea L.) cultivars. *Plant Physiol Biochem* **60**, 1-11 (2012).

162. Guerfel M, Baccouri O, Boujnah D, Chaïbi W, Zarrouk M. Impacts of water stress on gas exchange, water relations, chlorophyll content and leaf structure in the two main Tunisian olive (Olea europaea L.) cultivars. *Scientia Horticulturae* **119**, 257-263 (2009).

163. Brilli F*, et al.* Leaf and ecosystem response to soil water availability in mountain grasslands. *Agricultural and forest meteorology* **151**, 1731-1740 (2011).

164. Kakani VG, Vu JC, Allen LH, Boote KJ. Leaf photosynthesis and carbohydrates of CO 2-enriched maize and grain sorghum exposed to a short period of soil water deficit during vegetative development. *J Plant Physiol* **168**, 2169-2176 (2011).

165. Boussadia O, Mariem FB, Mechri B, Boussetta W, Braham M, El Hadj SB. Response to drought of two olive tree cultivars (cv Koroneki and Meski). *Scientia horticulturae* **116**, 388-393 (2008).

166. Koundouras S, Tsialtas IT, Zioziou E, Nikolaou N. Rootstock effects on the adaptive strategies of grapevine (Vitis vinifera L. cv. Cabernet–Sauvignon) under contrasting water status: leaf physiological and structural responses. *Agriculture, Ecosystems & Environment* **128**, 86-96 (2008).

167. Erice G, Louahlia S, Irigoyen JJ, Sánchez-Díaz M, Alami IT, Avice J-C. Water use efficiency, transpiration and net CO 2 exchange of four alfalfa genotypes submitted to progressive drought and subsequent recovery. *Environ Exp Bot* **72**, 123-130 (2011).

**Figure S1.** The relationship between the response ratios (lnRR) of the transpiration rate (T_r_) and growing temperature.


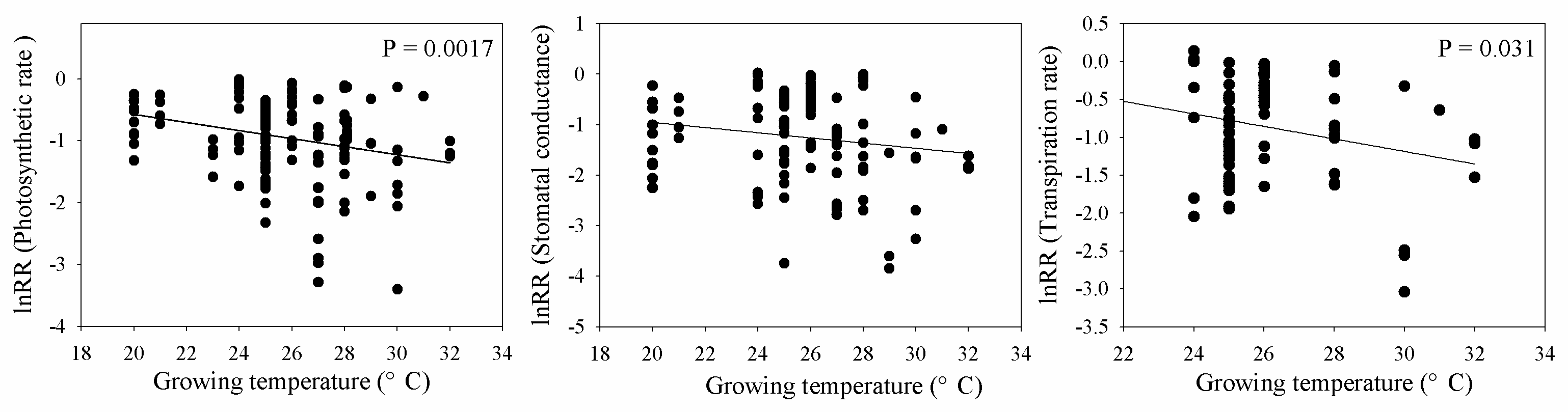


Figure S1
